# ELR510444 Inhibits Tumor Growth and Angiogenesis by Abrogating HIF Activity and Disrupting Microtubules in Renal Cell Carcinoma

**DOI:** 10.1371/journal.pone.0031120

**Published:** 2012-01-25

**Authors:** Jennifer S. Carew, Juan A. Esquivel, Claudia M. Espitia, Christoph M. Schultes, Marcel Mülbaier, Joe D. Lewis, Bernd Janssen, Francis J. Giles, Steffan T. Nawrocki

**Affiliations:** 1 Department of Medicine and Institute for Drug Development, Cancer Therapy and Research Center at The University of Texas Health Science Center, San Antonio, Texas, United States of America; 2 Elara Pharmaceuticals GmbH, Heidelberg, Germany; National Cancer Institute, United States of America

## Abstract

**Background:**

Hypoxia-inducible factor (HIF) is an attractive therapeutic target for renal cell carcinoma (RCC) as its high expression due to the loss of von Hippel-Lindau (VHL) promotes RCC progression. Considering this, we hypothesized that ELR510444, a novel orally available small molecule inhibitor of HIF activity, would reduce angiogenesis and possess significant activity in RCC. The mechanism of action and therapeutic efficacy of ELR510444 were investigated in *in vitro* and *in vivo* models of RCC.

**Principal Findings:**

ELR510444 decreased HIF-1α and HIF-2α levels, reduced RCC cell viability and clonogenic survival, and induced apoptosis. VHL-deficient RCC cells were more sensitive to ELR510444-mediated apoptosis and restoration of VHL promoted drug resistance. Higher concentrations of ELR51044 promoted apoptosis independently of VHL status, possibly due to the microtubule destabilizing properties of this agent. ELR510444 significantly reduced tumor burden in the 786-O and A498 RCC xenograft models. These effects were associated with increased necrosis and apoptosis and inhibition of angiogenesis.

**Conclusions:**

ELR510444 is a promising new HIF inhibitor that reduced RCC cell viability, induced apoptosis, and diminished tumor burden in RCC xenograft models. ELR510444 also destabilized microtubules suggesting that it possesses vascular disrupting and anti-angiogenic properties. Further investigation of ELR510444 for the therapy of RCC is warranted.

## Introduction

Overexpression of the hypoxia inducible factors (HIFs) HIF-1α or HIF-2α is associated with cancer progression [1], [2], [3], [4], [5], [6]. HIF-1 is a heterodimer composed of HIF-1α and HIF-1β subunits and HIF-2 consists of HIF-2α and HIF-1β subunits. HIF-1β or aryl hydrocarbon nuclear translocator (ARNT) is constitutively expressed and HIF activity is regulated by the expression of the α subunits [7]. The development of new blood vessels from the pre-existing vasculature (angiogenesis) is an essential process required for cancer progression. Under low oxygen conditions, the consequential upregulation of HIFs promote the increased expression of genes involved in angiogenesis (vascular endothelial growth factor, VEGF), metabolism (Glut-1), drug resistance (MDR-1), and cell survival (Bcl-2) [8], [9], [10]. Strategies that inhibit angiogenesis have become a viable therapeutic approach for many tumor types. VEGF is a major regulator of angiogenesis and antagonizing its function with the monoclonal antibody bevacizumab (Avastin) has demonstrated antitumor efficacy in preclinical models and in clinical trials [11], [12].

The multi-tyrosine kinase inhibitors sunitinib and sorafenib and the mTOR inhibitors temsirolimus/CCI-779 and everolimus/RAD001 have demonstrated efficacy for the treatment of renal cell carcinoma (RCC). The activity of these agents against RCC has been partially attributed to their ability to inhibit angiogenesis [12], [13]. Despite the success of these agents, drug resistance continues to be an obstacle, which underscores the need for new treatment strategies to improve clinical outcomes.

Mutations or loss of the von Hippel-Lindau (VHL) tumor suppressor gene are a frequent occurrence in RCC [14]. VHL is an E3 ubiquitin ligase that targets the α subunit of HIF for degradation via the proteasome. Loss of VHL expression results in the stabilization of HIFs and occurs in 70% of sporadic clear cell RCC patients [15]. In VHL-deficient cells, HIFs are constitutively active and induce target genes that promote tumor progression [16]. Consistent with the role of HIF in cancer, introduction of VHL into VHL-deficient RCCs suppresses tumor formation in mice [17]. Given this, targeting HIF activity may be a promising strategy to treat RCC and other malignancies with elevated HIF transcription rates.

ELR510444 is a novel, orally available small molecule HIF inhibitor that has been developed by ELARA Pharmaceuticals. Here we report that ELR510444 decreases HIF-1α and HIF-2α expression in RCC cells and cells deficient in VHL are hypersensitive to ELR510444-mediated apoptosis. ELR510444 also displayed significant efficacy in two RCC xenograft models *in vivo*. In addition, our findings demonstrate that higher concentrations of ELR510444 destabilize microtubules, suggesting that this agent has vascular disrupting properties. This is evident in tumors treated with ELR510444, which show enhanced central tumor necrosis along with diminished tumor vasculature.

## Materials and Methods

### Ethics Statement

This study was carried out in strict accordance with the recommendations in the Guide for the Care and Use of Laboratory Animals of the National Institutes of Health. The protocol (93088×) was approved by the Institutional Animal Care and Use Committee (Assurance #A3345-01) at the University of Texas Health Science Center at San Antonio.

### Cells and cell culture

786-O, A498, Caki-1, Caki-2, and Achn renal cancer cell lines were obtained from the American Type Culture Collection (Manassas, VA). RCC4 cells were obtained from Dr. Sunil Sudarshan (University of Texas Health Science Center at San Antonio). Cell lines were cultured in RPMI supplemented with 10% fetal bovine serum and maintained in a humidified incubator at 37°C with 5% CO_2_.

### Antibodies and reagents

Antibodies were obtained from the following commercial sources: anti-VEGF (Santa Cruz Biotechnology, Santa Cruz, CA); anti-HIF-1α (R&D Systems, Minneapolis, MN); anti-HIF-2α (Novus Biologicals, Littleton, CO); anti-cleaved caspase-3 (Cell Signaling, Danvers, MA); anti-β-tubulin (Sigma-Aldrich, St Louis, MO); anti-CD31 (BD Pharmingen, San Jose, CA); anti-proliferating cell nuclear antigen (PCNA) (Dako, Glostrup, Denmark); goat anti-rabbit and goat anti-rat horseradish peroxidase (HRP)-conjugated secondary antibodies (Jackson Laboratories, West Grove, PA); Rat anti-mouse IgG2a-HRP (Serotec, Raleigh, NC); Goat anti-mouse Alexa Fluor 488 and 4′,6-diamidino-2-phenylindole (DAPI) (Invitrogen, Carlsbad, CA); and sheep anti-mouse-HRP and donkey anti-rabbit-HRP (Amersham, Pittsburgh, PA). ELR510444 was kindly provided by ELARA Pharmaceuticals GmbH (Heidelberg, Germany). Vincristine, 1-methyl-2-pyrrolidone, CoCl_2_, and propidium iodide (PI) were purchased from Sigma-Aldrich. *N,N′*-ethylene-bis(iodoacetamide) (EBI) was obtained from TRC Biomedical Research Chemicals (New York, ON, Canada). Solutol HS15 was purchased from BASF (Florham Park, NJ). Polyethylene glycol 400 was obtained from Hampton Research (Aliso Viejo, CA).

### Quantification of drug-induced cytotoxicity

Cell viability was assessed by 3-(4,5-dimethylthiazol-2-yl)-2,5-diphenyltetrazolium bromide (MTT) assay. RCC cells were seeded in 96-well plates at a density of 10,000 cells per well and allowed to attach overnight. Cells were treated with varying concentrations of ELR510444 for 72 h. Cell viability was determined by incubating the cells in MTT, dissolving MTT in dimethyl sulfoxide (DMSO), and measuring absorbance using a BioTek (Winooski, VT) microplate reader. The pro-apoptotic effects of ELR510444 following 24 h treatment were quantified by PI staining and fluorescence-activated cell sorting (FACS) analysis of sub-G_0_/G_1_ DNA content as previously described [18].

### Clonogenic survival assays

Cells were treated with 3, 10, or 30 nM ELR510444 for 24 h. After drug treatment, cells were washed twice in PBS followed by the addition of fresh media and incubated for 10 days in a humidified incubator at 37°C with 5% CO_2_. Colonies were then washed in PBS, fixed with methanol, and stained with crystal violet. Colonies were counted using an Alpha Innotech (San Leandro, CA) gel documentation system.

### Immunoblotting

RCC cells were treated with ELR510444, collected, and lysed for 1 h on ice in Triton X-100 lysis buffer (1% triton X-100, 150 mM NaCl, 25 mM Tris pH 7.5) with protease inhibitors. Approximately 50 µg of total cellular protein from each sample were separated by SDS-PAGE. Proteins were transferred to nitrocellulose membranes and blocked with 5% nonfat milk in a Tris-buffered saline solution containing 0.1% Tween-20 for 1 h. The blots were probed overnight with the indicated primary antibodies at 4°C, washed, and then probed with species-specific secondary antibodies coupled to HRP. Bands were detected by enhanced chemiluminescence (Alpha Innotech, San Leandro, CA).

### HIF-1α activity assay

RCC4 cells were treated for 16 h with various concentrations of ELR510444. Cells were harvested and lysed on ice using HEPES lysis buffer (10 mM HEPES pH 7.9, 1.5 mM MgCl_2_, 10 mM KCl, 0.5 mM DTT) for 30 min followed by nuclear extraction (20 mM HEPES pH 7.9, 25% glycerol, 450 mM NaCl, 1.5 mM MgCl_2_, 0.2 mM EDTA pH 8.0, 0.5 mM DTT) for 30 min. HIF-1α activity was measured using the HIF-1α transcription factor assay kit (Cayman Chemical, Ann Arbor, MI) according to the manufacturer's instructions. Absorbance was quantified at 450 nm using a Biotek microplate reader.

### Quantitative real time polymerase chain reaction (qRT-PCR)

RCC4, 786-O and A498 cells were treated with 10 nM ELR510444 for 24 h. cDNA from Control and ELR510444 treated cells were used for relative quantification by RT–PCR analyses. First-strand cDNA synthesis was performed from 1 µg RNA in a 20 µl reaction mixture using the high-capacity cDNA Reverse Transcription Kit (Applied Biosystems, Foster City, CA). *HIF-1α*, HIF-2α (*EPAS1*), and *GAPDH* transcripts were amplified using commercially available TaqMan Gene expression assays (Applied Biosystems, Foster City, CA). Relative gene expression was calculated with the 2^−^ 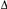 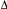 ^Ct^ method using *GAPDH* as a housekeeping gene [19].

### VEGF ELISA assay

VEGF secretion was measured in RCC cells using Quantikine ELISA kits (R&D Systems, Inc., Minneapolis, MN). Cells were plated in 6-well plates and were untreated, treated with 10 nM ELR510444 or 250 µM CoCl_2_ for 16 h. Supernatants were collected and VEGF protein levels were determined by ELISA according to the manufacturer's instructions. Absorbances were measured using a BioTek microplate reader.

### VHL transfection into RCC cells

Transfection of VHL into VHL-deficient cells was performed using the pCMV6-VHL-AC-GFP plasmid. The plasmid is a GFP-tagged ORF clone of *Homo sapiens* VHL, transcript variant 1 (OriGene, Rockville, MD) and transformed into One Shot TOP10 chemically competent *E. coli* (Invitrogen, Carlsbad, CA). After overnight incubation at 37°C, a single colony was grown in LB broth with ampicillin. The plasmid was isolated using the Qiagen mini-prep plasmid isolation kit (Qiagen Inc., Valencia, CA) and stored at −20°C until ready for transfection. A498 and 786-0 cells were transfected with 1 µg of pCMV6-VHL-AC-GFP and the empty vector using the TransFast reagent (Promega, Madison, WI) according to the manufacturer's protocol. Positive transfected clones were selected by serial dilution in the presence of puromycin.

### Immunocytochemistry

RCC4 cells were plated on chamber slides and treated with ELR510444 or 100 nM vincristine for 24 h. Cells were fixed with 4% paraformaldehyde, permeabilized using 0.2% Triton X-100, and incubated overnight with β-tubulin antibody. Goat anti-mouse Alexa 488 fluorescent secondary antibody was used to visualize microtubules. DAPI was used to counterstain the nucleus. Images were captured using a Zeiss LSM 510 Meta confocal microscope with an oil 40× objective as previously described [20].

#### Colchicine-binding site assay

*N,N′*-ethylene-bis(iodoacetamide) (EBI) crosslinks with the cysteine residues of β-tubulin at positions 239 and 354, which are associated with the colchicine-binding site [21]. The occupancy of the colchicine-binding site by antimitotic drugs, such as ELR510444 disrupts EBI: β-tubulin adduct formation. The β-tubulin adduct formed migrates faster than β-tubulin and is detectable by immunoblotting [22]. RCC4 cells were incubated with ELR510444 for 2 h prior to the addition of 100 µM EBI treatment for 1.5 h. After drug treatment, cells were processed for immunoblotting and probed with a β-tubulin antibody.

### Xenograft studies

786-O and A498 RCC cells (1×10^7^) were suspended in a mixture of HBSS and Matrigel (BD BioSciences, San Jose, CA) and subcutaneously implanted into female nude mice (BALB/c background) from Harlan (Indianapolis, IN). Tumor-bearing animals were randomized into vehicle or ELR510444 treatment groups. Mice were treated orally with vehicle (10% 1-methyl-2-pyrrolidone, 20% Solutol HS15, 40% polyethylene glycol 400, 30% water) or 8 mg/kg ELR510444 on a QDx5 (every day for 5 days) schedule for 2 weeks. Mice were monitored daily and tumor volumes were measured twice weekly. Tumors were harvested at the end of the study and either formalin-fixed and paraffin-embedded or OCT-embedded and snap-frozen for immunohistochemical analysis.

### Immunohistochemistry

Paraffin-embedded tumor sections were deparaffinized in xylene, exposed to a graded series of alcohol, and rehydrated in PBS (pH 7.5). Frozen slides were fixed by exposure to cold acetone, chloroform and acetone, and acetone again. Heat-induced epitope retrieval on paraffin-embedded sections was performed by microwaving slides in a citrate buffer for 5 min. Endogenous peroxides were blocked with a 3% hydrogen peroxide solution for 10 min. Slides were then incubated in a protein block solution (5% horse and 1% goat serum in PBS) for 20 min. PCNA, cleaved caspase-3, VEGF (paraffin-embedded tumors) or CD31 (frozen slides) antibodies were diluted in the protein block solution and placed at 4°C overnight. After washing with PBS, slides were incubated in appropriate secondary antibodies for 1 h at ambient temperature. Positive reactions were visualized using 3,3′-diaminobenzidine diaminobenzidine (Research Genetics, Huntsville, AL) for 10 min. The slides were rinsed with water followed by a brief counterstain with Gill's hematoxylin (Sigma, St. Louis, MO). Images were captured using an Olympus fluorescent microscope (Center Valley, PA) with a DP71 camera and a 20× objective. Image-Pro Plus software Version 6.2.1 (MediaCybernetics, Bethesda, MD) was used for image acquisition. ImageJ software was used for quantification of VEGF levels by densitometric analysis of five random fields containing viable tumor cells. Quantification of PCNA, cleaved caspase-3, and CD31 was conducted by counting the number of positive cells in five random fields.

### CD31 and Terminal deoxyribonucleotide-transferase–mediated dUTP nick-end labeling (TUNEL) assay

Frozen tumor sections were stained with CD31 antibody. Vessels were visualized by using a Texas Red Goat anti-Rat secondary antibody. DNA fragmentation was analyzed using a FITC-labeled TUNEL assay kit (Promega, Madison, WI) and performed according to the manufacturer's instructions. Slides were mounted using Prolong anti-fade reagent (Molecular Probes, Eugene, OR). Images were captured with an Olympus fluorescent microscope (Center Valley, PA) with a DP71 camera and a 20× objective. TUNEL-positive cells were determined by manual counting of 5 random fields per section. Image-Pro Plus software Version 6.2.1 (MediaCybernetics, Bethesda, MD) was used for image acquisition.

### Statistical analyses

The Tukey-Kramer Comparison Test or the Student's *t* test was used to determine statistical significance. Differences were considered significant in all experiments at p<0.05.

## Results

### ELR510444 reduces the expression of HIF-1α, HIF-2α, and VEGF in RCC cells

**Figure 1:**
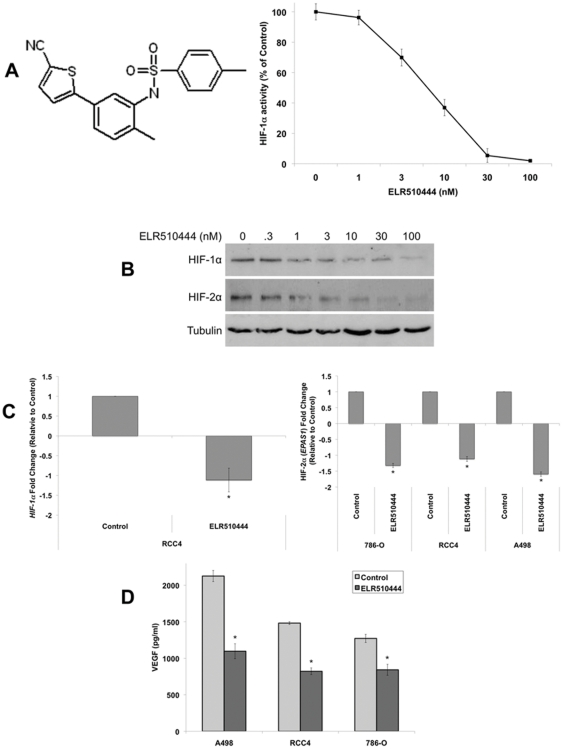
ELR510444 decreases HIF-1α and HIF-2α expression. (A) ELR510444 (chemical structure on left) inhibits HIF-1α activity. RCC4 cells were treated with the indicated concentrations of ELR510444 for 16 h. Cells were harvested, nuclei isolated and lysed, and HIF-1α activity was measured using a commercial activity kit according to the manufacturer's protocol. Mean ± SD, *n* = 3. (B) ELR510444 decreases HIF-1α and HIF-2α protein expression. RCC4 cells were treated with ELR510444 for 24 h and protein levels were measured by immunoblotting. (C) Quantitative real-time PCR analysis of *HIF-1α* and HIF-2α (*EPAS1*) levels. RCC cells were treated with 10 nM ELR510444 for 24 h and gene expression was measured by qRT-PCR. Mean ± SD, *n* = 3. *Indicates a significant difference from controls, p<0.05. Note that HIF-1α is not present 786-O and A498 cells. (D) ELR510444 decreases VEGF secretion. RCC cells were treated with 10 nM ELR510444 for 16 h. Media was harvested and VEGF levels were measured by ELISA. Mean ± SD, *n* = 3. *Represents a significant difference compared to controls, p<0.05.

HIFs play a key role in tumor progression and are an ideal target to inhibit angiogenesis. ELR510444 is a novel, orally available, small molecule inhibitor of HIF activity (Figure 1A). Treatment with ELR510444 inhibited HIF-1α activity at low nM concentrations in the VHL-deficient RCC4 cell line (Figure 1A). Further analysis demonstrated that ELR510444 induced a dose-dependent decrease in both HIF-1α and HIF-2α protein expression in RCC4 cells (Figure 1B). 786-O and A498 RCC cells also lack a functional VHL gene, but unlike RCC4 cells which express both HIF-1α and HIF-2α, 786-O and A498 cells express only HIF-2α [23]. Quantitative real-time PCR (qRT-PCR) analysis also revealed a significant decrease in HIF-1α levels in RCC4 cells and HIF-2α levels in all 3 VHL-deficient cell lines following ELR510444 treatment (Figure 1C). Since VEGF is a major transcriptional target of HIFs, we next evaluated the effects of ELR510444 on VEGF secretion. Consistent with inhibition of HIF activity, ELR510444 significantly decreased VEGF levels in RCC cell lines (Figure 1D).

### ELR510444 preferentially induces apoptosis in VHL-deficient RCC cells

**Figure 2:**
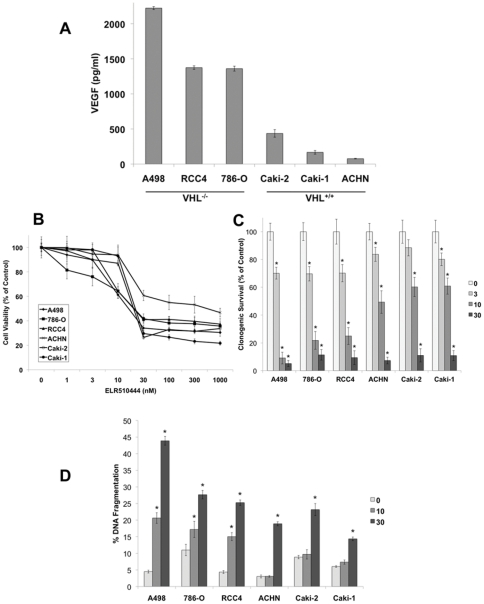
ELR510444 exhibits enhanced sensitivity in VHL-deficient RCC cell lines. (A) Quantification of VEGF levels in RCC cell lines. Equal numbers of cells were plated and media was collected 16 h later. VEGF levels were measured by ELISA assay. Mean ± SD, *n* = 3. (B) ELR510444 reduces RCC cell viability. Cells were treated with the indicated concentrations of ELR510444 for 72 h and viability was determined by MTT assay. Mean ± SD, *n* = 3. (C) ELR510444 reduces clonogenic survival. RCC cells were treated with the indicated concentrations of ELR510444 for 24 h. After treatment, cells were washed in fresh media and allowed to grow for 10 days. Colonies were fixed and stained with crystal violet and quantified using an Alpha Innotech gel imager. Mean ± SD, *n* = 3. *Indicates a significant difference compared to controls, p<0.05. (D) ELR510444 induces apoptosis. Cells were treated with ELR510444 for 24 h and apoptosis was measured by PI-FACS analysis. Mean ± SD, *n* = 3. *Denotes a significant difference compared to controls, p<0.05.

**Figure 3:**
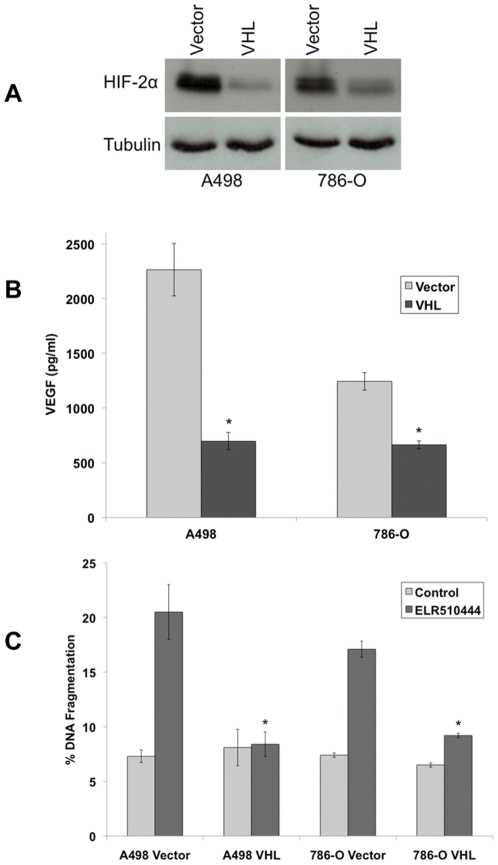
VHL-deficient cells are hypersensitive to ELR510444-induced apoptosis. (A) HIF-2α is reduced in A498 and 786-O cells transfected with VHL. Cells were transfected with empty vector or VHL. Positively transfected cells were selected by incubation with puromycin. HIF-2α was measured by immunoblotting. (B) VEGF levels are reduced in the presence of VHL. Cells were plated and media was collected 16 h later. VEGF levels were measured by ELISA assay. Mean ± SD, *n* = 3. *Indicates a significant difference compared to controls, p<0.05. (C) A498/VHL and 786-O/VHL cells are less sensitive to ELR510444-mediated apoptosis. Cells were treated with 10 nM ELR510444 for 24 h and apoptosis was measured by PI-FACS analysis. Mean ± SD, *n* = 3. *Represents a significant difference compared to ELR510444-treated vector-only transfected cells, p<0.05.

Loss of VHL expression is linked to constitutive HIF activity and consequentially high levels of VEGF expression. Quantification of VEGF levels in VHL^−/−^ and VHL^+/+^ RCC cell lines confirmed the relationship between VHL status and VEGF (Figure 2A). Considering the proposed regulatory role of HIF in RCC tumorigenesis and drug resistance, we hypothesized that targeting HIF with ELR510444 would be an effective strategy for the treatment of RCC. To test our hypothesis, we first evaluated the activity of ELR510444 in a panel of RCC cell lines. MTT and clonogenic survival assays demonstrated that ELR510444 exhibited strong activity against all of the RCC lines tested (Figure 2B and 2C). In addition to reducing cell viability and colony formation, ELR510444 also stimulated apoptosis in RCC cell lines (Figure 2D). Interestingly, ELR510444 possessed greater efficacy against VHL-deficient cancer cells compared to VHL^+/+^ cell lines. This effect was most pronounced at the concentration of 10 nM ELR510444, which preferentially induced apoptosis in VHL^−/−^ cell lines (Figure 2D). These data suggested that ELR510444 may be more efficacious in cell lines with elevated HIF activity. To investigate this possibility, we transfected wild-type VHL into the A498 and 786-O VHL-null cell lines. As expected, introduction of VHL into these cell lines resulted in a reduction in HIF-2α expression (Figure 3A) and VEGF secretion (Figure 3B). We next evaluated the sensitivity of mock (vector only) and VHL transfected cell lines to ELR510444. Introduction of VHL into A498 and 786-O cells significantly reduced ELR510444-mediated apoptosis (Figure 3C). Collectively, these data demonstrate that VHL-deficient RCC cells are preferentially sensitive to ELR510444 treatment.

### ELR510444 inhibits hypoxia-induced expression of HIF-2α

**Figure 4:**
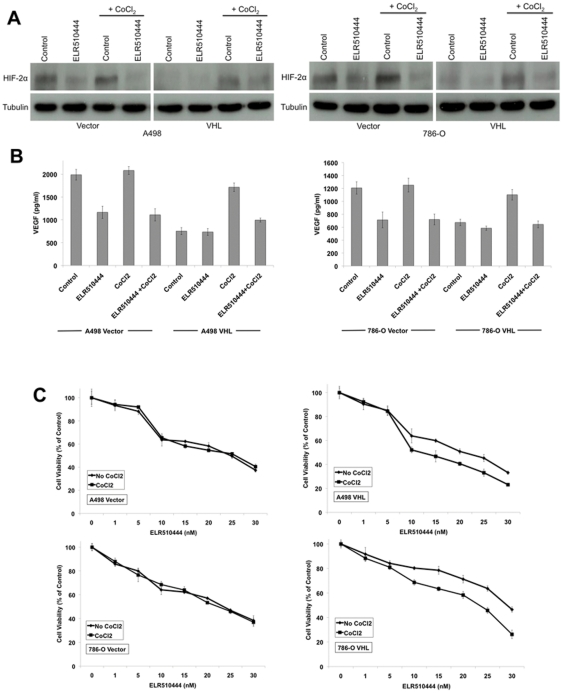
ELR510444 antagonizes CoCl_2_-induced HIF and VEGF expression and has activity under hypoxic conditions. (A) HIF-2α is reduced in A498 and 786-O cells treated with ELR510444. Vector- and VHL-transfected cells were treated with 10 nM ELR510444 in the presence or absence of 250 µM CoCl_2_ for 24 h. HIF-2α was measured by immunoblotting. (B) VEGF levels are reduced by ELR510444. Cells were plated and treated with 10 nM ELR510444 ± 250 µM CoCl_2_. 16 h later, media was collected and VEGF levels were measured by ELISA assay. Mean ± SD, *n* = 3. (C) A498/VHL and 786-O/VHL cells are more sensitive to ELR510444 under hypoxic conditions. Cells were treated with the indicated concentrations of ELR510444 for 72 h ± 250 µM CoCl_2_. Cell viability was measured by MTT assay. Mean ± SD, *n* = 3.

The effect of hypoxia on the activity of ELR510444 was investigated in vector- and VHL-transfected A498 and 786-O cells. Cobalt chloride (CoCl_2_) was used to mimic hypoxia. As expected, the addition of CoCl_2_ stimulated HIF-2α expression in the VHL-transfected cells (Figure 4A). Importantly, ELR510444 effectively reduced HIF-2α levels under both basal and hypoxic conditions (Figure 4A). ELR510444 also reduced VEGF secretion under hypoxic conditions and levels corresponded to HIF-2α expression (Figure 4B). The effect of ELR510444 on cell viability was next evaluated in vector- and VHL-transfected cell lines under hypoxic conditions. The addition of CoCl_2_ did not alter the sensitivity of vector-transfected cells (VHL^−/−^) to ELR510444 (Figure 4C). However, stimulation of hypoxia enhanced ELR510444's activity in the VHL-transfected cells, suggesting that ELR510444 may be more effective against cells with high HIF activity (Figure 4C).

### ELR510444 depolymerizes microtubules and arrests RCC cells in mitosis

**Figure 5:**
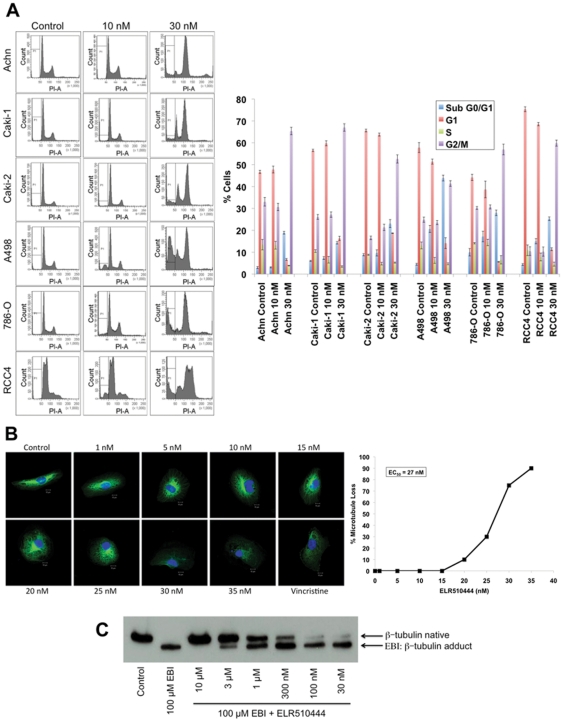
ELR510444 arrests RCC cells in mitosis and depolymerizes microtubules. (A) RCC cells were treated with 10 and 30 nM ELR510444 for 24 h. Cell cycle distribution was determined by PI-FACS analysis. Representative histograms and the percentage of cells in each phase of the cell cycle are shown for each experimental condition. (B) RCC4 cells were plated on chamber slides and treated for 24 h with the indicated concentrations of ELR510444 or 100 nM vincristine. Cells were stained with a β-tubulin antibody and microtubules were visualized using an Alexa Fluor 488-conjugated secondary antibody. Nuclei were counterstained using DAPI. The percent microtubule depolymerization was determined at each concentration visually in 100 cells. Representative images are shown. (C) ELR510444 interacts with the colchicine-binding site of β-tubulin. RCC4 cells were pre-treated with the indicated concentrations of ELR510444 for 2 h. 100 µM EBI was then given for an additional 1.5 h. Cells were harvested for immunoblotting and blots were probed with a β-tubulin antibody to detect native β-tubulin and EBI: β-tubulin adducts.

While 10 nM ELR510444 induced apoptosis preferentially in VHL-null RCC cells, higher concentrations (30 nM–1000 nM) stimulated apoptosis in RCC cells regardless of VHL status (Figure 5A and data not shown). Analysis of cell cycle distribution following treatment with 30 nM ELR510444 revealed that apoptosis induction was associated with mitotic arrest (Figure 5A). Due to the prominent M phase arrest that we observed, we hypothesized that ELR510444 may have tubulin binding properties. Immunocytochemistry revealed that ELR510444 administered at concentrations as low as 20 nM and the majority of cells at 30 nM exhibited microtubule destabilization (Figure 5B), which is a hallmark feature of many agents that disrupt tumor vasculature. The microtubule-destabilizing drug vincristine was used as a positive control. To further evaluate the microtubule-binding properties of ELR510444, we investigated whether this agent interacts with the colchicine-binding site. Using a method based on the ability of the chemical EBI to form adducts with β-tubulin, we determined that ELR510444 interacts with the colchicine-binding site by occupying this site and preventing EBI: β-tubulin adduct formation (Figure 5C). Taken together, the effects of ELR510444 on HIF expression and microtubule destabilization suggest that ELR510444 possesses both anti-angiogenic and vascular disrupting properties.

### ELR510444 reduces tumor burden in RCC xenografts

**Figure 6:**
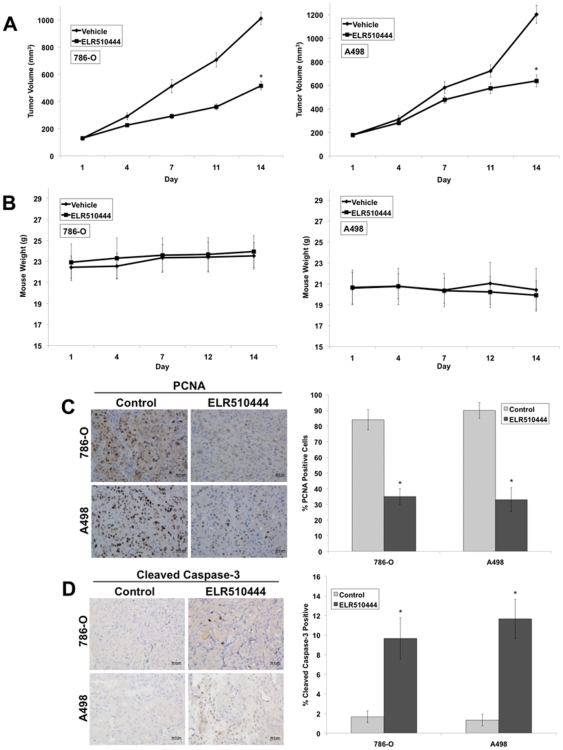
ELR510444 reduces tumor burden and cell proliferation and stimulates apoptosis. (A) 786-O and A498 cells (1×10^7^/mouse) were injected into the flanks of nude mice. When tumors reached approximately 150 mm^3^ in size, mice were randomized into groups and treated on a QDx5 schedule with 8 mg/kg ELR510444 for 2 weeks. Tumor volume was measured twice weekly. Mean ± SEM, *n* = 10. *Indicates a significant difference compared to vehicle, p<0.05. (B) ELR510444 was well tolerated *in vivo*. Animal body weight was measured throughout the study to quantify drug-induced weight loss. Mean ± SD, *n* = 10. (C) ELR510444 reduces tumor cell proliferation. PCNA levels were determined by conducting immunohistochemistry on tumor sections. Positive cells were scored manually. Mean ± SD, *n* = 5. *Indicates a significant difference compared to controls (vehicle), p<0.05. (D) ELR510444 induces apoptosis. Apoptosis was measured by cleaved caspase-3 staining. Quantification was conducted by manually counting positive cells. Mean ± SD, *n* = 5. *Indicates a significant difference compared to controls (vehicle), p<0.05.

To further investigate the anticancer activity of ELR510444, we evaluated its efficacy in the 786-O and A498 RCC mouse xenograft models. 786-O and A498 tumor-bearing animals were randomized into groups and given 8 mg/kg ELR510444 orally for 2 weeks on a QDx5 (every day for 5 days) schedule. Treatment with ELR510444 significantly decreased mean tumor volume in both xenograft models compared to the vehicle-treated controls (Figure 6A). Importantly, ELR510444 was very well tolerated as no significant animal weight loss was observed throughout the duration of the study (Figure 6B). Further analysis of tumors harvested at the end of the study revealed a significant reduction in tumor cell proliferation as measured by PCNA staining (Figure 6C) and an increase in cleaved caspase-3 levels, a marker of apoptosis (Figure 6D). Collectively, these data demonstrate that ELR510444 has significant *in vivo* activity in RCC tumor models.

### Inhibition of angiogenesis and enhanced central tumor necrosis are associated with the antitumor activity of ELR510444

**Figure 7:**
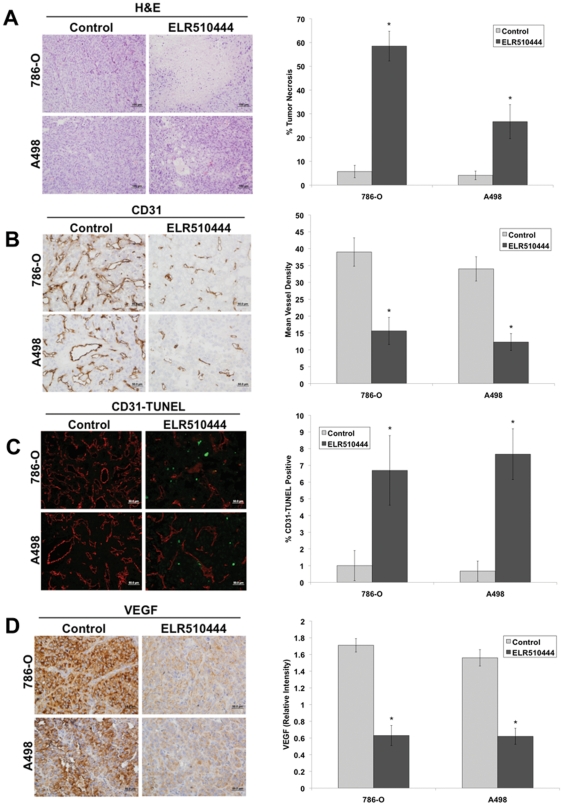
ELR510444 increases central tumor necrosis and inhibits angiogenesis. (A) ELR510444 induces tumor necrosis. Tumor sections were stained with H&E to visualize necrosis. Percent necrosis was calculated using ImageJ software. Mean ± SD, *n* = 5. *Indicates a significant difference compared to controls (vehicle), p<0.05. (B) ELR510444 reduces microvessel density. Immunohistochemistry was performed using CD31 antibody to visualize vessels. Mean vessel number was quantified manually in 5 independent sections. *Represents a significant difference compared to controls (vehicle), p<0.05. (C) ELR510444 induces endothelial cell death. Tumor sections were stained with CD31 followed by TUNEL assay. CD31-TUNEL-positive cells were counted manually. Mean ± SD, *n* = 5. *Indicates a significant difference compared to controls (vehicle), p<0.05. (D) Quantification of VEGF levels by immunohistochemistry. The relative intensity of VEGF expression was measured using Image-Pro Plus software Version 6.2.1. Mean ± SD, *n* = 5. *Denotes a significant difference compared to controls, p<0.05.

Our *in vitro* studies demonstrated that ELR510444 reduced HIF-1α and HIF-2α expression and destabilized microtubules. We next investigated the effects of ELR510444 treatment on central tumor necrosis, a marker of vascular disruption, and tumor angiogenesis. Hematoxylin and Eosin (H&E) staining revealed significantly elevated central tumor necrosis in both the 786-O and A498 tumors treated with ELR510444 (Figure 7A). Immunohistochemistry was performed to evaluate the impact of ELR510444 on microvessel density. ELR510444 treatment significantly reduced mean vessel density as measured by CD31 staining (Figure 7B). Dual staining of CD31 and TUNEL determined that ELR510444 stimulated the apoptosis of tumor endothelial cells (Figure 7C). Consistent with our *in vitro* data, ELR510444 treatment led to a strong decrease in VEGF levels (Figure 7D). Our data demonstrate that ELR510444 inhibits angiogenesis, disrupts tumor vasculature, and induces apoptosis in RCC xenograft models.

## Discussion

HIFs are frequently overexpressed in a broad range of tumor types, including renal, breast, and head and neck cancers and often correlate with poor clinical prognosis [1], [2], [3], [4], [5], [6], [16], [24], [25]. Since elevated HIF expression promotes tumor progression, inhibition of HIF activity is a promising approach to treat cancer. This strategy may be especially effective against RCCs that have constitutive HIF activity due to the loss of VHL expression. Consistent with this idea, temsirolimus and everolimus, which are both approved for RCC therapy, have been shown to reduce HIF activity by blocking mTOR activation [26], [27]. Other agents with anti-angiogenic activity including sunitinib and vorinostat have also displayed an ability to reduce HIF expression and possess efficacy against RCC [27], [28]. While these agents have displayed activity in RCC, drug resistance is a significant problem, thus highlighting the need for new therapies.

Several other agents that have been identified as HIF-1α inhibitors including YC-1, PX-12, and PX-478 have exhibited efficacy in both *in vitro* and *in vivo* tumor models, further validating HIF as a therapeutic target [29], [30], [31]. Our results show that ELR510444 decreases HIF-1α and HIF-2α expression, inhibits angiogenesis, and depolymerizes microtubules via interaction with the colchicine-binding site on β-tubulin. ELR510444 also demonstrated promising activity in RCC xenografts *in vivo*, which was associated with decreased angiogenesis and enhanced necrosis. The ability of this agent to decrease both HIF-1α and HIF-2α levels combined with its anti-angiogenic and anti-vascular properties suggest that it may have significant efficacy against highly vascularized tumors such as RCC. Vascular-directed therapies that target both angiogenesis and the pre-existing vasculature may yield enhanced antitumor activity as compared with agents that specifically target new or intrinsic vessels. Consistent with this idea, an earlier study demonstrated that co-treatment of bevacizumab with the vascular disrupting agents combretastatin A4 phosphate (Zybrestat) or combretastatin A1 phosphate (OXi4503) produced significantly improved anticancer activity compared to single agent therapy [32].

Investigation of the efficacy of ELR510444 in a panel of RCC cell lines revealed that it displayed preferential efficacy in RCC cells lacking functional VHL. Furthermore, introduction of wild-type VHL into VHL-deficient cells conferred resistance to ELR510444-mediated apoptosis. Interestingly, a previously conducted study demonstrated that VHL-deficient cells were also more sensitive to the mTOR inhibitor temsirolimus/CCI-779 [33]. These observations indicate that cells lacking VHL may be hypersensitive to agents that block HIF activity. Since inactivation of VHL is frequently observed in RCC, it may be possible to exploit this phenomenon therapeutically.

In addition to decreasing HIF expression, ELR510444 also depolymerizes microtubules at higher concentrations. The ability of ELR510444 to disrupt microtubules has recently been reported in other cell types as the drug was found to bind to the colchicine-binding site of β-tubulin, which is similar to other vascular disrupting agents such as the combretastatins [34]. Our data supports this finding as we also show that ELR510444 interacts with the colchicine-binding site of β-tubulin in RCC cells. This suggests that ELR510444 targets the pre-existing vasculature as well as new vessel formation. Similar to ELR510444, 2-methoxyestradiol (2-ME) decreases HIF-1α and HIF-2α levels and depolymerizes microtubules [35], [36]. 2-ME has demonstrated activity against various cancer models, but displayed limited bioavailability in clinical trials [37], [38], [39]. Analogs of 2-ME designed to improve its stability and efficacy are currently in development.

Recent work has described HIF-independent functions of VHL demonstrating that it is a microtubule-binding protein that promotes microtubule stability [40]. Further studies showed that VHL inhibited nocodazole-induced microtubule depolymerization, reduced microtubule turnover, and enhanced stability by decreasing mitotic catastrophe [40], [41]. Since VHL promotes microtubule stability, VHL-deficient cells may be more prone to cell death induced by ELR510444 or other microtubule depolymerizing agents. It is possible that this activity also contributes to the efficacy of ELR510444 in VHL-null RCC.

ELR510444 is a promising new agent that warrants further investigation for the treatment of RCC and other malignancies that display elevated HIF activity. Collectively, our data establish that ELR510444 decreases HIF-1α and HIF-2α expression, inhibits angiogenesis, possesses vascular disrupting properties, and provide the foundation for future clinical investigation of ELR510444.
